# Pigmented Full-Thickness Human Skin Model Based on a Fibroblast-Derived Matrix for Long-Term Studies

**DOI:** 10.1089/ten.tec.2021.0069

**Published:** 2021-07-16

**Authors:** Patrícia Zoio, Sara Ventura, Mafalda Leite, Abel Oliva

**Affiliations:** ^1^ Biomolecular Diagnostic Laboratory, Instituto de Tecnologia Química e Biológica (ITQB), Universidade Nova de Lisboa (UNL), Oeiras, Portugal.; ^2^ Department of Biomolecular Diagnostics, Instituto de Biologia Experimental e Tecnológica (IBET), Oeiras, Portugal.

**Keywords:** skin, full thickness, tissue engineering, fibroblast-derived matrix, fully human, transepithelial electrical resistance

## Abstract

Reconstructed human skin models are a valuable tool for drug discovery, disease modeling, and basic research. In the past decades, major progress has been made in this field leading to the development of full-thickness skin models (FTSms) better representative of the native human skin by including the cellular cross talk between the dermal and epidermal layers. However, current available FTSms still present important limitations since they are only suitable for short-term studies, include nonhuman extracellular matrix (ECM) components and have a weak skin barrier function compared with *in vivo* human skin. In this study, a fibroblast-derived matrix was combined with the use of an inert polystyrene scaffold for the development of a fully human dermis capable of supporting a differentiated epidermis. To produce a pigmented FTSm, a coculture with keratinocytes, melanocytes, and fibroblasts was established. The structure and functionality of the developed FTSms were studied for short- and long-term cultivation using histological and immunofluorescence staining. The integrity of the skin barrier was evaluated using transepithelial electrical resistance (TEER) measurements. It was possible to obtain a mature dermis capable of supporting an epidermis without keratinocyte infiltration in only 6 days. ECM components (collagen IV and fibrin) were secreted by the fibroblasts and accumulated in the scaffold structure, recreating the microenvironment of the native human dermis. Moreover, the use of a scaffold resulted in a structure with mechanical stability due to its noncontracting nature. The coculture of primary human keratinocytes resulted in a terminally differentiated skin equivalent that could maintain its architecture and homeostasis up to 50 days. Melanocytes were correctly integrated within the epidermal basal layer and made it possible to reproduce constitutive pigmentation. TEER levels increased during culture time, reaching values of 1.1 ± 0.2 kΩ.cm^2^ for the FTSm, indicative of a functional skin barrier.

**Impact statement:**

The developed fully human full-thickness skin model has the potential to reduce the dependence on animal models for long-term studies of skin diseases and safety and efficacy assessment of novel drugs. Its longevity and robustness allow the experimental testing phase to be lengthened. The presence of active melanocytes at the dermal–epidermal junction makes this model the ideal platform to study skin pigmentation disorders.

## Introduction

In recent years, there has been an increase in the demand for physiologically relevant *in vitro* human skin models for evaluating the safety and efficacy of new drug formulations or cosmetic ingredients and for studying skin biology and skin-related diseases. This need has been further amplified by European Union regulations that encourage the 3Rs concept, calling for Replacement, Reduction, and Refinement of animal experimentation and bans the use of animals for testing active compounds for cosmetics (Cosmetics regulation EC No. 1223/2009).^1^

The majority of the available skin models are reconstructed human epidermis models (RHEms) generated by seeding keratinocytes on a porous membrane. Although these models provide highly standardized conditions for drug testing, they do not include the cellular epidermal–dermal cross talk, which limits its applicability.^2^ More recently, full-thickness skin models (FTSms) have been developed by seeding keratinocytes onto a dermal compartment composed of fibroblasts embedded in a matrix. Most FTSms are generated using collagen-based hydrogels, usually of bovine or rat-tail origin.^3,4^ Although these hydrogels offer a good support for epidermis formation, they are susceptible to fibroblast-mediated contraction during culture, resulting in excessive deformation.^5,6^ The high contractibility of the dermal matrix renders the models unsuitable for long-term studies. To overcome this, solutions based on chemically cross-linking the dermal layer or creating more stable composites have been proposed. In particular, Vidal *et al.* presented a new matrix consisting of a silk-collagen composite system, which reduced contraction and degradation over time.^7^ Lotz *et al.* prolonged the skin longevity by using succinimidyl glutarate polyethylene glycol to cross-link cell seeded collagen.^8^ However, most of these systems include animal-derived components and are not able to completely inhibit contraction. Matrices including nonhuman extracellular matrix (ECM) components introduce batch-to-batch variability and are not representative of the healthy *in vivo* human skin microenvironment, which contains lipids, fibrin, glycosaminoglycans, and proteoglycans.^9^

An alternative approach for the generation of FTSm is based on the use of human fibroblast-derived matrices (FDMs) as dermal equivalents. For this, multilayered human fibroblasts are stimulated to generate ECM by modulation of various culture conditions. This approach results in a stratified FTSm with higher concentration of natural moisturizing factors and longer lifespan compared with animal collagen-based skin equivalents.^10^ However, a major disadvantage of using FDMs is the requirement for extended culture periods to produce sufficient quantities of ECM ranging from weeks to months due to the lack of a scaffold.^11,12^ Also, some features such as thickness and mechanical stability of the FDMs introduce high variability.^13^

Hill *et al.* generated a mechanically stable three-dimensional (3D) microenvironment mimicking the native skin by seeding fibroblasts on an inert porous scaffold, enabling the formation of an FDM with defined thickness.^14^ This model was successfully used to investigate early melanoma invasion within a fully human microenvironment. However, one of the main limitations of using scaffolds for generating FDMs is the potential for keratinocyte infiltration into the dermis resulting in the absence of a continuous epidermal layer and epidermis disorganization. Considering this, Roger *et al.* studied the optimized timeframe for dermis formation.^15^ Although the group could generate a skin model that recapitulates various features of *in vivo* skin, their protocol still required >40 days to produce the FTSms.

We report the development of a robust and stratified FTSm in a total of 21 days. By seeding a high density of human dermal fibroblasts on a porous scaffold and stimulating the cells to produce FDM, a dermal equivalent capable of supporting an epidermis is produced in only 6 days. The resultant FTSm is highly differentiated and closely mimic aspects of the architecture of the *in vivo* skin tissue. Moreover, a pigmented version of the FTSm is produced by introducing melanocytes. A complex structure with coculture with three different skin cell types is obtained, ideal to study pathologies that require advanced.

## Methods

### Primary cells and cell maintenance

Primary human dermal fibroblasts isolated from neonatal foreskin (HDFn; CellnTec) were maintained in fibroblast growth medium (FGM) composed of Iscove's modified Dulbecco's medium (Gibco^®^, Waltham, MA) supplemented with 10% fetal bovine serum ( Gibco). Primary human epidermal keratinocytes isolated from neonatal foreskin (HEKn; Gibco) were maintained in keratinocyte growth medium (KGM) composed of EpiLife medium (Gibco), supplemented with 0.06 mM calcium and keratinocyte growth factor (HKGS, Gibco). Primary human melanocytes (HEM; Gibco) were maintained in melanocyte growth medium (MGM) composed of M254 medium (Gibco) supplemented with melanocyte growth factor (HMGS, Gibco). All cells were maintained at 37°C and 5% carbon dioxide (CO_2_) in a humidified incubator. This study only included commercially available cells. It was approved by the iNOVA4Heath platform (UID/Multi/04462/2013) and followed the research ethical rules of the Universidade Nova de Lisboa.

### Generation of the FTSm

**FIG. 1.**
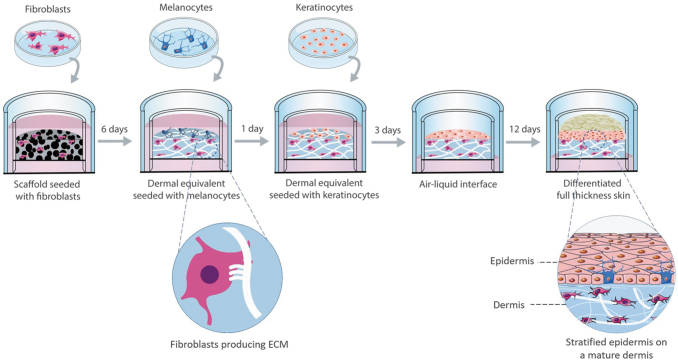
Schematic representation of the developed methodology for pigmented FTSm generation using coculture with three cell types (HDFns, HEKns, and HEMs). The process can be divided in four main steps: the development of a mature dermis, the culture of HEMs on top of the dermis, the culture of epidermal cells under submerged conditions, and the culture of the epidermis at the ALI until a fully differentiated skin is generated. After 12 days, a stratified epidermis on top of a mature dermis is obtained. ALI, air–liquid interface; FTSms, full-thickness skin models; HDFns, human dermal fibroblasts isolated from neonatal foreskin; HEKns, human epidermal keratinocytes isolated from neonatal foreskin; HEM, human melanocytes. Color images are available online.

The process of developing full-thickness and fully human skin can be divided in the main steps depicted in Figure 1: the generation of a mature dermis, the expansion of keratinocytes on top of the dermis under submerged conditions and the development of the epidermis at the air–liquid interface (ALI). Fully human dermal equivalents were generated by seeding HDFns within inert porous polystyrene scaffolds (Alvetex^®^, REPROCELL Europe Ltd., United Kingdom) with void sizes of 33–55 μm and 90% porosity. This makes them the ideal structure for the HDFns to penetrate and lay down FDM. In brief, scaffolds were pretreated with 70% ethanol to render them hydrophilic followed by two washes with phosphate-buffered saline (PBS). 1.0 × 10^6^ HDFs were seeded onto the scaffolds in 100 μL FGM and incubated at 37°C, in a humidified atmosphere of 5% CO_2_ for 1.5 h. After the cell adhesion period, FGM supplemented with 100 μg/mL l-ascorbic acid 2-phosphate (Sigma-Aldrich) was applied to the bottom of each well to flood the insert before incubation and maintained up to a further 18 days at 37°C in a 5% CO_2_ humidified incubator, changing the media every 3 days, to study the formation of the dermal equivalent.

Full-thickness human skin models were generated by seeding 5.0 × 10^5^ HEKn cells onto the dermal equivalents. HEKns were incubated under submerged conditions in KGM containing high calcium concentration (1.5 mM) and maintained at 37°C, in a humidified atmosphere of 5% CO_2_. After 3 days, the models were raised to ALI by removal of the medium in the upper compartment and cultured in KGM containing 1.5 mM calcium, supplemented with 10 ng/mL KGF and 50 μg/mL of l-ascorbic acid 2-phosphate. The FTSms were maintained up to a further 50 days.

### Generation of the pigmented FTSm

Pigmented FTSms were developed by seeding 5.0 × 10^4^ HEMs on top of the dermis compartment. The tissues were incubated in MGM under submerged conditions at 37°C and 5% CO_2_ for cell adhesion. After 24 h, 5.0 × 10^5^ HEKns were seeded on top of the dermis and HEM layer. The cells were maintained in coculture growth medium (CGM) composed of 80% KGM containing high calcium (1.5 mM) and 20% MGM under submerged conditions. After 3 days, ALI was established and the models were maintained in CGM supplemented with 10 ng/mL KGF and 50 μg/mL of l-ascorbic acid 2-phosphate for 12 days.

### Generation of an RHEm on a polycarbonate membrane

Cell culture inserts with polycarbonate membranes (Merck Millipore) were used for RHEm generation. The polycarbonate membranes were selected for HKEn attachment without matrix. In brief, the inserts were placed in six-well plates containing 2.5 mL KGM with high calcium concentration (1.5 mM) and seeded with 1.5 × 10^5^ HEKns in 500 μL FGM medium. After 24 h, the models were raised to ALI. The medium was replaced by 1.5 mL KGM supplemented with 1.5 mM calcium, 50 μg/mL l-ascorbic acid 2-phosphate, and 10 ng/mL KGF, and renewed every other day.

## Experiments

### Experimental design

#### Strategy

The presented method was used to produce fully human dermal equivalents with different maturation times. FTSms were generated using the developed dermal equivalents and maintained for up to 50 days. The morphology and functionality of the produced FTSm were evaluated and compared with an *in house* RHEm. This was performed through histological and immunofluorescence analysis and through transepithelial electrical resistance (TEER) measurements. For each experiment, an *N* = 3 and *n* = 3 were used.

#### Histological analysis and immunohistochemistry

The reconstructed tissues were fixed immediately after being taken out of culture in 10% neutral buffered formalin (Sigma-Aldrich). The samples were embedded in paraffin to allow for transverse sectioning. Paraffin-embedded sections were deparaffinized and rehydrated for morphological evaluation by staining with hematoxylin and eosin (H&E) through standard methods.

Skin sections (5-μm-thick) were mounted on slides and incubated during 30 min in a dry chamber. Sections were stained with anti-ki67 (Roche), anti S-100 (Roche), and anti-tyrosinase (Roche) primary antibodies diluted in a solution of PBS containing 1% bovine serum albumin (PBS-BSA 1%; BSA, Sigma-Aldrich).

#### Immunofluorescence analysis

Skin sections were deparaffinized using HistoChoice^®^ (Sigma-Aldrich) and incubated in a dry chamber at 65°C during 30 min. Then, skin sections were hydrated during 5 min in a gradient of ethanol solutions (100%, 96%, and 70%). The antigen retrieval was performed by heating in citrate buffer (pH = 6). For anti-collagen IV antibody, the antigen retrieval was followed by proteinase K (20 μg/mL in TE buffer) incubation for 30 s at 37°C with 10 min of cooling at room temperature. Immunofluorescence staining of sections was performed using the following primary antibodies: 1:500 anti-collagen IV (Abcam), 1:10 anti-cytokeratin 10 (Progen), 1:1000 anti-cytokeratin 14 (Abcam), 1:100 anti-cytokeratine 15 (Sigma-Aldrich), 1:250 anti-fibronectin (Abcam), 1:50 anti-filaggrin (Invitogen), and 1:100 anti-collagen I (Abcam) diluted in a solution with PBS-BSA 1% with overnight incubation in humidified chamber at 4°C. Secondary antibodies, 1:1000 Alexa Fluor^®^ 488 Goat Anti-Rabbit IgG (Invitrogen) or 1:1000 FITC Goat Anti-Mouse IgG (Sigma-Aldrich), diluted in a solution with PBS-BSA 1%, were then added with an incubation of 2 h. Nuclei were stained with 1 μg/mL of 4,6-diamidino-2-phenylindole (DAPI, Invitrogen) and slides were mounted with Vectashield (Vector Laboratories). Images were obtained using the Nikon Eclipse TE2000-S fluorescence microscope (Nikon instruments, USA) and analyzed with the ImageJ Software.

#### TEER evaluation

The barrier integrity of developed models was evaluated by TEER. Measurements were taken using an Epithelial Volt/Ohm Meter (EVOM) and a pair of Ag/AgCl probes (WPI Europe, United Kingdom). Measurements were performed during 14 days of cell culture for FTSm and RHEm. TEER values were calculated according to the following equation:

$$TEER=\left(R_{sample}-R_{blank}\right)\times A,$$

where *R_sample_* is the resistance value for the skin model, *R_blank_* is the resistance value of an insert without cultured cells, and *A* the effective culture area (1.12 cm^2^ for FTSm and 0.63 cm^2^ for RHEm). For the RHEms, the KGM was replaced with 300 μL PBS in the apical region and 2 mL PBS in the basal region. For FTSm, for the time required to measure electrical resistance, the tissues were placed in an adapted six-well plate containing 2 mL PBS. The KGM was aspirated and replaced with 250 μL of PBS in the apical region. For both models, the probe was placed such that one electrode was submerged in the upper compartment and the other was submerged in the lower compartment. TEER values were recorded during culture time from the last day under submerged conditions (day 0) until day 14 at ALI. These measurements were performed for three batches of three skins at each time point for FTSm and RHEm.

### Experimental results

#### Generation of a mature dermis capable of supporting a differentiated epidermis

**FIG. 2.**
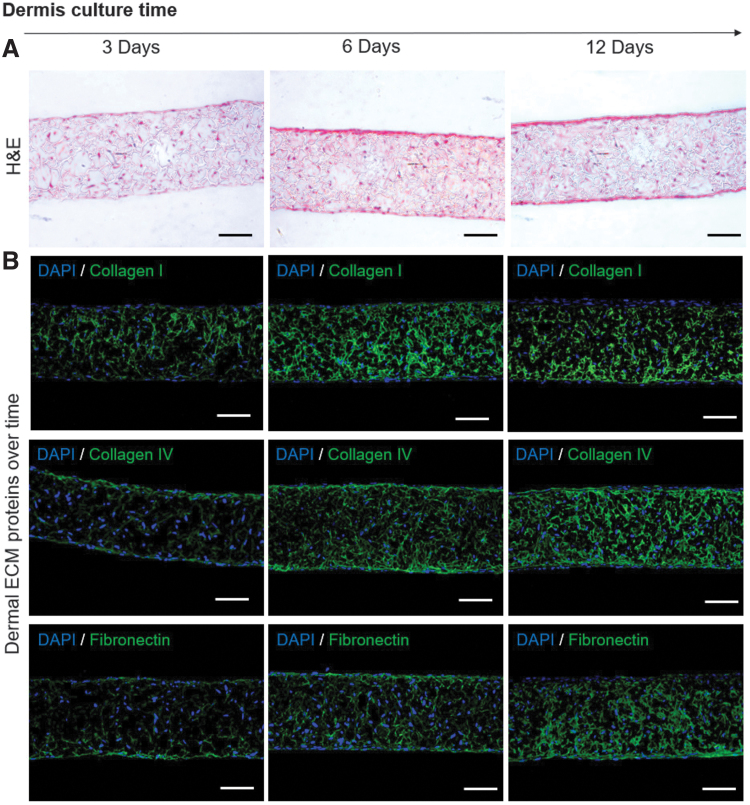
HDFns seeded on inert porous scaffolds and stimulated to produce ECM for 3, 6, and 12 days **(A)** Representative H&E images of dermal models cultured over time. **(B)** Representative immunofluorescence images showing ECM proteins (collagen I, collagen IV, and fibronectin) deposited on the dermal models cultured over time. Scale bars: 100 μm. ECM, extracellular matrix; H&E, hematoxylin and eosin. Color images are available online.

The first step to obtain an organized FTSm was the optimization of the culture times to produce a mature dermal equivalent capable of supporting the epidermis and minimize keratinocyte infiltration. As can be seen in Figure 2A, dermal HDFns cultured for 3–12 days in the porous scaffold show a homogeneous distribution within the scaffolds. HDFns are stimulated to produce FDM, which build up and accumulate inside the scaffold. This can be seen in Figure 2B, which shows ECM markers expressed in the dermal construct from 3 to 12 days of. HDFn culture. Collagen I, collagen IV, and fibronectin were detected within the dermal equivalents since day 3 of culture. An increase in the color intensity could be observed over time, which shows an increase in quantities of FDM being produced by the HDFns and being deposited within the scaffold structure.

**FIG. 3.**
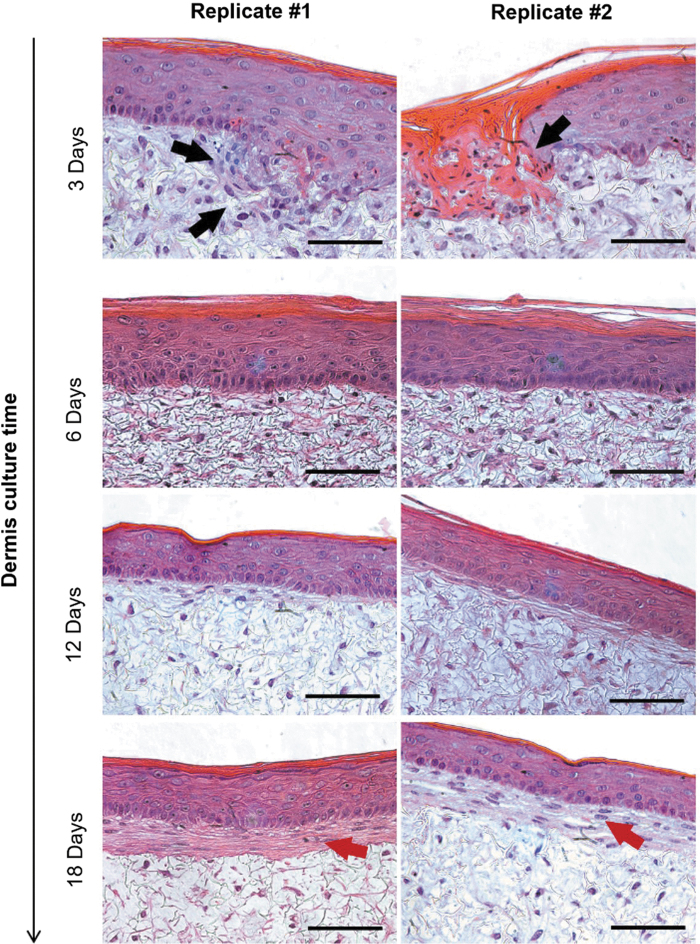
Representative H&E images of two independent replicates of FTSm (HDFns + HEKns) developed using dermis with different maturation times (from 3 to 18 culture days) and maintained at ALI for 12 days to promote epidermal differentiation. *Black arrows* point to keratinocyte infiltration on a dermis cultured for 3 days. *Red arrows* point to ECM deposition on *top* of the scaffold on a dermis cultured for 18 days. Scale bars: 100 μm. Color images are available online.

The influence of the dermis maturation on the epidermis formation was evaluated by seeding HEKns on top of dermal equivalents cultured for different times. Figure 3 shows cross sections of FTSm generated using dermal constructs matured from 3 to 18 days. Epidermis grown on a 3-day dermal construct shows keratinocyte infiltration, resulting in areas of epidermal disorganization. From 6 to 18 days of dermis formation, a differentiated and organized epidermis is achieved. No signs of keratinocyte infiltration can be seen in these constructs. During culture time, a layer of fibroblasts and ECM starts to deposit at the basal and apical sides of the scaffold, reaching 50 ± 15 μm thickness at day 33 of culture (data not shown).

#### FTSm resembling *in vivo* human skin morphology and homeostasis

**FIG. 4.**
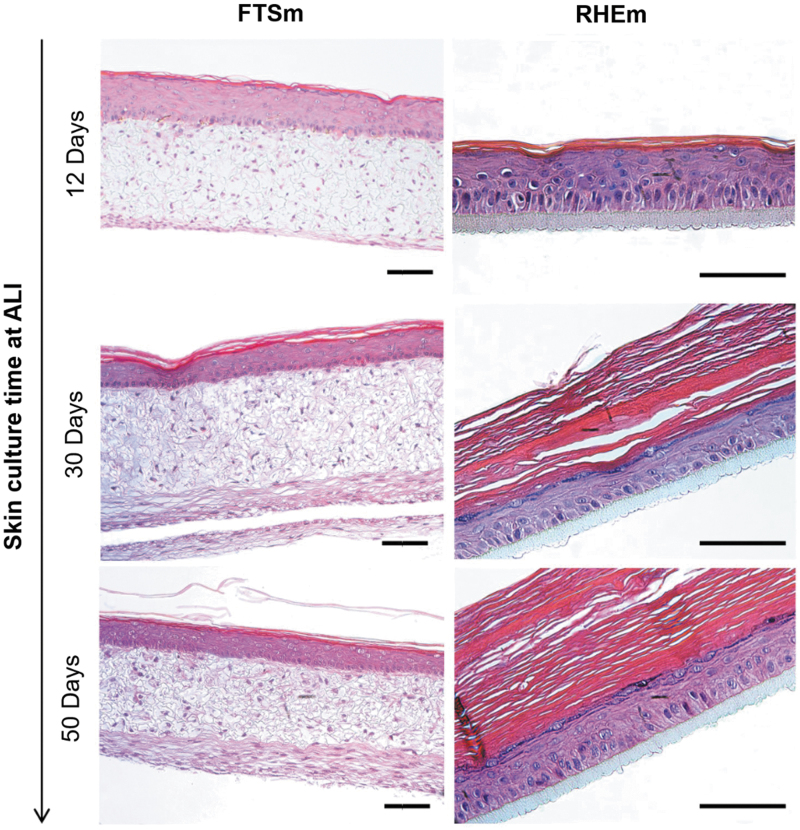
Representative H&E images of skin models (FTSm and HEKns) maintained over time at ALI (up to 50 days). For the FTSm (HDFns + HEKns) a 6-day dermis was used. Scale bars: 100 μm. Color images are available online.

The morphology of the FTSm was evaluated and compared with the RHEm (Fig. 4). After 12 days at ALI, both FTSm and RHEm displayed native human skin-like morphology, including a well-organized and stratified epidermis. H&E staining reveals all layers of the epidermis: stratum basale (SB), stratum spinosum (SS), stratum granulosum (SG), and stratum corneum (SC).

FTSm and RHEm were kept at ALI for up to 50 days and its structure was monitored over time, confirming the suitability of the developed model for long-term studies. Histological sections after a cultivation time of 30 days show a differentiated epidermis, including the presence of all layers characteristic of the *in vivo* human epidermis. Although there is a reduction in epidermal thickness when compared with the models cultured for 12 days at ALI, the pattern of uniform cuboid basal keratinocytes and preserved architecture indicates the model maintains their functionality. From day 30 to 50 of culture at ALI, both RHEm and FTSm remained stable, maintaining the same thickness and identifiable epidermal layers. For RHEm, the reduction in thickness of the lower cellular layers was accompanied by an increase in cornified acellular upper layers, with the SC reaching a thickness of ∼200 ± 25 μm at day 50 of culture. FTSm cultured long term did not show an increase in the SC thickness.

**FIG. 5.**
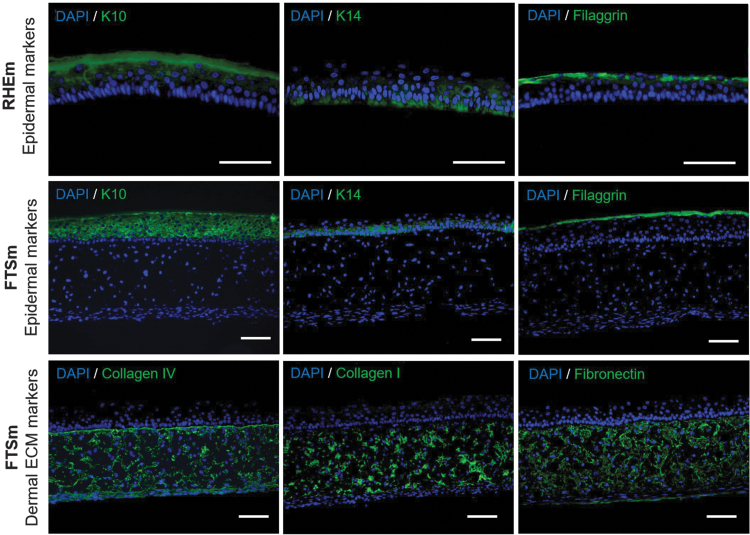
Immunofluorescence analysis of FTSm (HDFns + HEKns) and RHEm. The FTSm was generated by culturing the dermal compartment for 6 days, then allowing epidermal differentiation for 12 days ALI. The RHEm was generated by allowing epidermal differentiation at ALI for 12 days. Data show protein expression patterns for key biomarkers typically associated with epidermal differentiation (K10, K14, and filaggrin) and with ECM compounds found in the dermis (collagen I, collagen IV, and fibronectin). Scale bars: 100 μm. RHEm, reconstructed human epidermis model. Color images are available online.

**FIG. 6.**
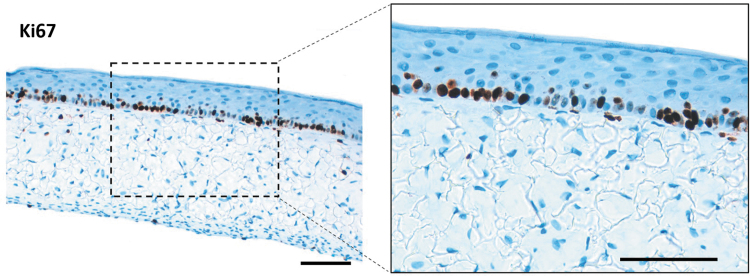
Analysis of HEKn proliferation during FTSm formation. Immunohistological staining performed with Ki67, showing proliferative activity in the basal layer. Scale bars: 100 μm. Color images are available online.

The presence and distribution of protein biomarkers representing various stages of epidermal differentiation was assessed by immunofluorescence staining in FTSm and RHEm (Fig. 5). Keratin (K) 14, an intermediate filament in the SB of the epidermis, was expressed in the SB and immediate suprabasal layer. K10 was expressed and distributed in the SS layers and extended to all suprabasal layers. These two markers were similarly expressed in FTSm and RHEm. Filaggrin, a terminal differentiation marker, was correctly deposited at the SG and SC layers for the FTSm and RHEm. This demonstrates the differentiated nature of the models. Collagen IV, a major component of the lamina densa, was present within the dermis and showed an increase staining intensity along the dermo-epidermal junction of the FTSm. Collagen I and fibronectin, major components of the dermis, presented a homogenous distribution within this compartment. Proliferative activity of HEKns was identified by immunohistological staining with Ki67, a protein accumulated within the nucleus during mitosis. The results show that the FTSm was actively proliferating through mitosis with Ki67-positive cells restricted to the SB (Fig. 6). This is expected since, on *in vivo* skin, the stem and progenitor cells reside on the SB, undergoing self-renewing or differentiative cell divisions.

#### FTSm with active melanocytes resembling *in vivo* pigmentation

**FIG. 7.**
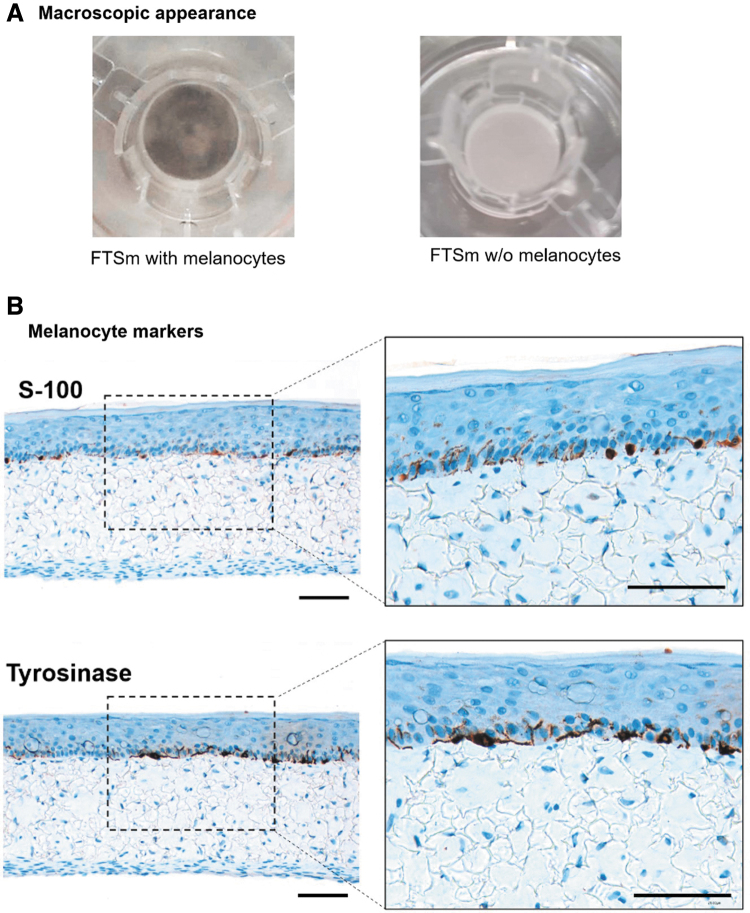
Examination and measurement of pigmentation in the developed FTSm after 6 days of dermis formation and 12 days at ALI for epidermis differentiation. **(A)** Macroscopic appearance of the FTSm with melanocytes (*left*) in comparison with FTSm without melanocytes (*right*). *Left* picture shows the pigmentation caused by the presence of melanocytes **(B)** Representative immunohistochemistry sections evaluating S-100 protein (*top*) and tyrosinase (*bottom*) to detect the presence of melanocytes in the epidermal compartment. Scale bars: 100 μm. Color images are available online.

After 12 days at ALI, a stratified epidermal structure with HEMs correctly integrated was achieved. Macroscopically, the presence of pigmentation on the FTSm can be clearly seen when compared with a FTSm without added HEMs (Fig. 7A). The macroscopic pictures show pigmentation across the culture area with decreased intensity at the center. The presence of melanocytes in the epidermis was evaluated by S-100 protein staining (Fig. 7B, top) on FTSm cross sections, revealing their localization in the basal cell layer interspersed with basal cell HEKns. In addition, tyrosinase, a key enzyme for controlling the production of melanin, was identified through immunohistochemistry (Fig. 7B, bottom). Tyrosinase was highly expressed in the basal layer of the epidermis.

#### TEER as an indicator of the skin barrier formation

**FIG. 8.**
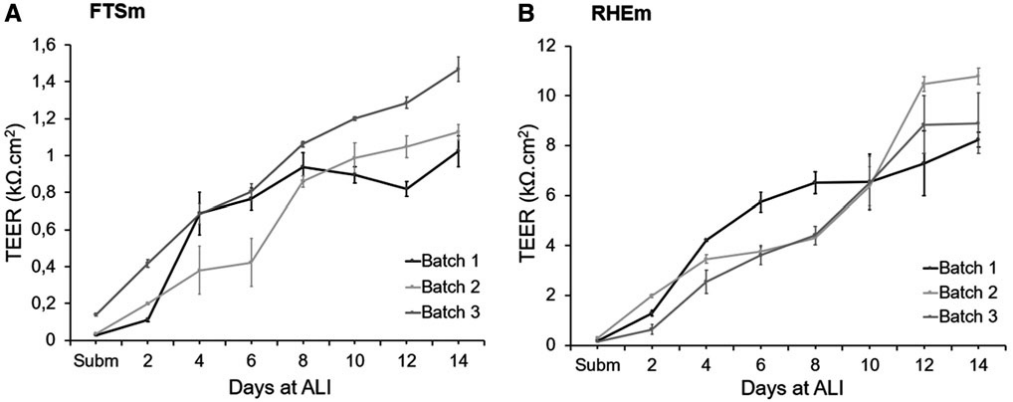
TEER measurements performed with an EVOM system and chopstick electrodes obtained from day 0 to day 12 at ALI for **(A)** FTSm (HDFns + HEKns) and **(B)** RHEm. For the FTSm, a 6-day dermis was used. Data represent the average TEER of three biological replicates and for three technical replicates at each time point. Reported TEER as Ω.cm^2^ tissue surface area. Error bars represent mean ± standard deviation. EVOM, Epithelial Volt/Ohm Meter; TEER, transepithelial electrical resistance.

The barrier integrity of the stratifying cultures was evaluated by TEER, measured during skin culture from day 0 (submerged conditions) until day 14 at ALI for RHEm and FTSm (Fig. 8). For both models, TEER values under submerged conditions started below 0.2 kΩ.cm^2^ and, as the epidermis gradually developed, increased significantly reaching values of 1.1 ± 0.2 kΩ.cm^2^ for FTSm (Fig. 8A) and 9.3 ± 1.1 kΩ.cm^2^ for RHEm (Fig. 8B).

## Discussion

We present a fully human FTSm based on an FDM with an *in vivo*-like architecture and optimized coculture with three different skin cell types (fibroblasts, melanocytes, and keratinocytes), compatible with long-term cultivation. The developed FTSm includes a mechanically stable dermal compartment with a physiological 3D microenvironment, a fully differentiated epidermis resembling the structure and function of the native counterpart as well as pigmentation by including active melanocytes at the basal layer. Moreover, we evaluate the suitability of using TEER measurements as a nondestructive technique to quantify in real-time the barrier integrity of FTSm during the various stages of skin differentiation.

The first goal was the development of a robust dermis that could reproduce the physiological matrix and microenvironment of the native human dermis and that could support a stratified epidermis. Adding a dermis to the skin model fits the need to better mimic the physiological architecture of human native skin since dermal fibroblasts interact with keratinocytes, playing a role in skin tissue morphogenesis, homeostasis, and histopathological conditions.^16^ Conventional FTSms using animal-derived hydrogels present several limitations, including batch variability, species-specific effects, and lack of mechanical stability due to fibroblast-mediated contraction during cell culture. These characteristics greatly diminish their applicability and negatively impact studies requiring topically application of substances^5^ and integration in various platforms such as organ-on-a-chip devices.^6,17^

We present an alternative approach where human fibroblasts are seeded in a porous inert scaffold and stimulated to produce an FDM resulting in a fully human dermal component that maintains their mechanical structure long term. In the developed dermis we could observe expression of collagen I, collagen IV, and fibronectin with accumulation of ECM with time. Previous studies using a similar technique needed ∼4 weeks to produce a mature dermis capable of supporting the epidermis, followed by an epidermal differentiation of at least 14 days.^15^ This long process greatly minimizes their applicability in industry and clinical contexts. In this study, we were able to reduce the necessary time for dermal formation to <1 week by optimizing the cell seeding density and medium composition. Contrary to conventional FTSm, the developed model shows no reduction of the epidermal surface area due to shrinkage, resulting in a more accurate model of the skin barrier. The 6-day dermis was able to support an epidermis without signs of keratinocyte infiltration and, at the same time, minimized the accumulation of a thick layer of fibroblasts and ECM on the apical and basal sides of the scaffold, which leads to a faster nutrient depletion from the cell culture medium.

It was possible to reproduce the complex multilayered structure present in healthy *in vivo* skin, with an observable stratified squamous epithelium structured in basal, spinous, granular, and corneal layers. This stratification is a critical feature of a relevant skin models and correlates with its barrier function. The developed FTSm reflected the expected expression pattern of keratins, with expression of K14 in the basal layer and K10 in the suprabasal layers. Filaggrin, a component of the cornified cell envelope, important for the structure and integrity of the SC, was correctly expressed in the FTSm. We show that the developed FTSm can maintain its viability and function for at least 50 days at ALI. Conventional FTSm not only contract over time but are also prone to degradation by matrix metalloproteases after 2 weeks.^18^ This extended lifespan compared with standard models makes it more suitable to study physiological and biochemical processes for a long period of time. Contrary to other long-term FTSm, the presented model does not include animal-derived hydrogels and does not require complex methods to generate a stable dermis that could hamper its reproducibility. Recently, given the increased interest in prolonging the experimental testing phase, a long-term skin model became commercially available (Phenion^®^ Full-thickness LONG-LIFE skin model). This model can be kept in culture for up to 50 days. However, these commercial systems lack flexibility since they arrive premade and the methods used to generate them are not transparent. With our open-source FTSm, it will be possible to study skin reactions taking multiple days to manifest or requiring repeated dose administration as well as recovery after tissue treatment.

Thickening of the SC during a prolonged culture period is a common phenomenon for *in vitro* skin models, indicating the accumulation of the corneocytes layer without proper desquamation.^19^ The gradual thickening of the SC indicates a lack of epidermal homeostasis. This phenomenon was observed in the developed RHEm, with the SC reaching a thickness of 200 ± 25 μm at day 50 of culture at ALI. Histological analysis of the FTSm showed an absence of SC accumulation with extended culture time. This observation can indicate an optimized epidermal homeostasis with a desquamation process mimicking the native skin.^20^ These results point to the potential of the FTSm to study mechanisms in epidermal regeneration and tissue homeostasis. Further studies should be performed to clarify this observation by analysis of the enzymatic activity in SC.

We also developed a pigmented version of the FTSm with active melanocytes at the dermo-epidermal junction. The pigmentation could be clearly seen macroscopically. This model could be of great interest for drug screening studies and disease modeling. Since drug-induced pigmentation accounts for ∼20% of all cases of hyperpigmentation, this model could be useful to study this side effect of new developed drugs.^21^

TEER analysis was used to evaluate the stage of skin differentiation and as a method of quality control. TEER reflects skin barrier functionality by measuring overall barrier to ions. It takes into account the contributions from the SC barrier and cell-to-cell tight junctions (TJ), which regulate the movement of ions across the paracellular pathway.^22^ During RHEm and FTSm culture for 2 weeks at ALI there is an increase in TEER values, reflecting progressive SC accumulation and TJ formation. RHEm reached values of 9.3 ± 1.1 kΩ.cm^2^ and FTSm reached values of 1.1 ± 2.3 kΩ.cm^2^. Although the FTSm does not experience contraction during cell culture, an increased collagen accumulation at the insert wall interface results in an area where keratinocyte adhesion is reduced. This explains why the TEER values are lower when compared with RHEm. In fact, it is documented that a tissue surface coverage of <99.6% causes an 80% drop in barrier function,^23^ resulting in dramatic decrease in TEER values. Still, TEER values for both FTSm and RHEm fall within the range of some validated *in vitro* skin models, including Epiderm™ (0.5 ± 0.1 kΩcm^2^)^24^ and other models without formal regulatory acceptance, including Keraskin™-VM (> 0.5 kΩcm^2^)^25^ and dermo–epidermal skin equivalents (> 1 kΩcm^2^).^26^

## Conclusion

We have developed an advanced FTSm, which, contrary to conventional techniques, does not include animal-derived components. In this model, human fibroblasts are stimulated to produce FDM resulting in a fully human structure that resembles the *in vivo* microenvironment. The use of an inert scaffold results in a noncontracting and stable structure, increasing reproducibility and applicability. The model exhibits *in vivo*-like characteristics, including a well-differentiated and organized epidermis. We showed the FTSm can be maintained for at least 50 days, making it ideal for long-term studies, for example, modeling melanoma and aging. Moreover, the potential of this model for achieving higher complexity is demonstrated by successfully reproducing skin pigmentation. In the future, other cell types could be introduced in the FTSm such as immune and endothelial cells to recreate a more advanced platform for disease modeling.
